# Comparative Genomics of the Transport Proteins of Ten *Lactobacillus* Strains

**DOI:** 10.3390/genes11101234

**Published:** 2020-10-21

**Authors:** Hassan Zafar, Milton H. Saier

**Affiliations:** 1 Department of Molecular Biology, Division of Biological Sciences, University of California, San Diego, La Jolla, CA 92093-0116, USA; 2 Department of Microbiology and Molecular Genetics, Faculty of Life Sciences, University of Okara, Okara, Punjab 56300, Pakistan

**Keywords:** *Lactobacillus*, transport proteins, probiotic, pathogenic

## Abstract

The genus *Lactobacillus* includes species that may inhabit different anatomical locations in the human body, but the greatest percentage of its species are inhabitants of the gut. Lactobacilli are well known for their probiotic characteristics, although some species may become pathogenic and exert negative effects on human health. The transportome of an organism consists of the sum of the transport proteins encoded within its genome, and studies on the transportome help in the understanding of the various physiological processes taking place in the cell. In this communication we analyze the transport proteins and predict probable substrate specificities of ten *Lactobacillus* strains. Six of these strains (*L. brevis*, *L. bulgaricus*, *L. crispatus*, *L. gasseri*, *L. reuteri*, *and L. ruminis*) are currently believed to be only probiotic (OP). The remaining four strains (*L. acidophilus*, *L. paracasei, L. planatarum, and L. rhamnosus)* can play dual roles, being both probiotic and pathogenic (PAP). The characteristics of the transport systems found in these bacteria were compared with strains (*E. coli*, *Salmonella*, and *Bacteroides*) from our previous studies. Overall, the ten lactobacilli contain high numbers of amino acid transporters, but the PAP strains contain higher number of sugar, amino acid and peptide transporters as well as drug exporters than their OP counterparts. Moreover, some of the OP strains contain pore-forming toxins and drug exporters similar to those of the PAP strains, thus indicative of yet unrecognized pathogenic potential. The transportomes of the lactobacilli seem to be finely tuned according to the extracellular and probiotic lifestyles of these organisms. Taken together, the results of this study help to reveal the physiological and pathogenic potential of common prokaryotic residents in the human body.

## 1. Introduction

In humans and other mammals, members of the genus *Lactobacillus* colonize the gastrointestinal tract (GIT), oral cavity and female genitourinary tract [1]. Members of this genus comprise a paraphyletic group of Gram-positive and non-spore-forming lactic acid bacteria with over 239 species and 29 subspecies. Most species are non-motile; however, some species like *Lactobacillus ruminis* may exhibit flagellar motility [2,3]. Generally, lactobacilli may be anaerobic or aerotolerant and can also assume the roles of commensals, probiotics, and opportunistic pathogens [4]. During glucose fermentation by lactobacilli, the main metabolic end product is lactic acid while acetic acid, succinic acid, ethanol, and carbon dioxide are also produced in small amounts [5]. Based on the types of fermentative pathways and their end metabolic products, lactobacilli can be classified into three groups: i) metabolic group A members (obligately homofermentative), which use glycolysis to ferment hexoses exclusively to lactic acid and are unable to ferment pentoses and gluconate, ii) metabolic group B members (facultatively heterofermentative), which use the glycolytic pathway to ferment hexoses to lactic acid, but also use an enzyme phosphoketolase (enzyme of the pentose phosphate pathway) to degrade pentoses and gluconate to acetic acid, formic acid and ethanol, and iii) metabolic group C members (obligate heterofermentative), which metabolize hexoses and pentose via the phosphogluconate pathway to produce lactic acid, acetic acid or ethanol and CO_2_ [6]. Overall, lactobacilli have a limited metabolic repertoire for the synthesis of essential nutrients, and consequently, they rely on other organisms or food sources to provide vitamins, amino acids, nucleic acid derivatives and sugars [7]. The extensive nutritional requirements of these microbes confine them to locations in the mammalian body that are rich in these required nutrients [8].

*Lactobacillus* spp. are considered to be efficient probiotic organisms in the GIT of humans [9]. The ability of these organisms to produce lactic acid and other metabolites helps to kill pathogenic microbes [10]. Among these metabolites there are antimicrobial proteins called bacteriocins; these are small proteins or peptides that are ribosomally synthesized and are effective at killing pathogenic microbes [11]. Other beneficial effects of lactobacilli include regulation of the immune system, maintenance of normal intestinal homeostasis, improvement of gastrointestinal barrier function and suppression of proinflammatory cytokines [12,13,14]. For further insight about the potential beneficial roles of lactobacilli, see [12].

Generally, *Lactobacillus* spp. assume the role of commensal and probiotic organisms in the gut. However, they can be opportunistic pathogens and cause a variety of infections such as abscesses, bacteremia, endocarditis, pulmonary infections, and neonatal meningitis [15]. Most of the disease conditions caused by lactobacilli occur in immunocompromised individuals or those that have predisposing conditions like diabetes. Further details about the possible mechanisms of disease mediated by lactobacilli have been elaborated in a review [16].

The research reported herein is in continuation of our project describing the transportome of the human gut bacteriome. This is the fourth edition in the series, as previously we have reported our findings about the physiological, metabolic, and pathogenic roles of transport proteins in *E. coli*, *Salmonella* and *Bacteroides* strains [17,18,19]. The strains analyzed in the previous projects included commensals, beneficial bacteria, and pathogens. In the current study, we analyzed the transportomes of ten strains (species) of *Lactobacillus.* Most of the strains are residents of the mammalian GIT, while some are members of the vaginal flora and oral cavity. All ten strains have been shown to exhibit host beneficial attributes; however, as noted above, strains may assume pathogenic roles in various regions of the human body.

For identification of the strains throughout this communication, the first letter from the genus *Lactobacillus* (L) will be denoted in capital, followed by the first two letters as lower-case from the species. Table 1 provides a general introduction to the ten *Lactobacillus* spp. examined. Some of the strains are only probiotic with no experimentally demonstrated pathogenicity; such strains are termed as only probiotic (OP) while strains that have both probiotic and pathogenic potential will be abbreviated as pathogenic and probiotic (PAP).

## 2. Materials and Methods

### 2.1. Genome-BLAST of the Ten Lactobacillus Proteomes

The FASTA formatted protein coding sequences of *L. acidophilus* NCFM [20], *L. brevis* ATCC 367 [21], *L. crispatus* ST1 [22], *L. delbrueckii* subsp. bulgarius ATCC 11,842 [23], *L. gasseri* ATCC 3323 [21], *L. paracasei* subsp. paracasei JCM 8130 [24], *L. plantarum* WCFS1 [25], *L. rhamnosus* GG [26], *L. ruminis* ATCC 27782 [27] and *L. reuteri* DSM 20016 (unpublished), were individually inputted into the GBLAST program [28]. The reason behind the selection of the lactobacilli strains was the qualities of the drafts, completeness, relevant health benefits and potential to cause diseases.

Initially, the proteomes were screened for potential homologs of proteins in the Transporter Classification Database (TCDB; www.tcdb.org) in March 2020 using GBLAST. For each protein, the program retrieves information for both the genome query and TC top hit sequence, the TC number of the latter, the numbers of amino acyl residues (aas) and numbers of predicted transmembrane segments (TMSs) in both proteins, the query to hit e-value, sequence similarity among regions, and TMS overlap in different regions between the queries and hits (proteins). The number of TMSs are predicted by GBLAST through the Web Based Hydropathy, Amphipathicity and Topology (WHAT) program. This program aligns the plots of hydrophobicity and amphipathicity through the length of the proteins (query and hit) [29,30]. However, proteins lacking TMSs were not omitted, as soluble components are often present in multicomponent systems that could be homologs of transport proteins.

### 2.2. Examination of Distant Transport Protein Homologues in Lactobacillus spp.

Initially, an arbitrary e-value cut off of 0.0001 was used for the GBLAST searches, followed by manual analysis of the proteins that had >0.0001 e-values. This examination based on topological data was done to determine the likelihood of the proteins to be either true homologues or false positives. As two proteins may give small e-values due to homology among hydrophilic regions, a manual examination of overlapping regions was necessary to prevent the selection of good scoring proteins that were not actually homologous in their transmembrane domains. The WHAT program-generated hydropathy profiles were used to determine whether the program had made incorrect TMS predictions. Furthermore, the AveHAS program was used for the confirmation of predicted proteins with homologues [30]. Proteins having e-values between 0.0001 and e^−8^, indicated a range in which there was a possibility of the presence of distant protein homologs. Consequently, these proteins were examined using the steps mentioned above.

### 2.3. Identification of Substrates Transported

According to TCDB hit entries, the predicted homologues were assigned substrate specificities. For this purpose, the proteins with unknown substrates were assigned substrates according to literature. In general, substrate assignments were justified depending on (1) the family to which the protein belongs, (2) the magnitude of the e-value, and (3) the region of greatest sequence similarity.

### 2.4. Analysis of Multicomponent Systems

Several multicomponent transport systems encoded within the *Lactobacillus* genomes were identified. If the transmembrane protein was found the system was considered to be functional in the strains. However, when possible, all constituents of such a system were sought, and these could sometimes be identified. This was possible because in prokaryotes, all constituents of such a multicomponent transport system are usually (but not always) encoded within a single gene cluster, and even within a single operon. This fact also facilitates assignment of probable substrate, because together with the transporter genes, related metabolic enzymes may also be encoded.

## 3. Results

### 3.1. Subclasses of Transport Proteins

The analysis of the occurrence of transporters in the ten lactobacilli was done using the methodologies described in the Methods section and our recent genomic publications [17,18,19]. For complete results of this study, see the Supporting information section (Supplementary Table S1). The distribution of subclasses of transport proteins identified in the tens strains is given in Table 2.

### 3.2. Channel Proteins (TC Subclass 1.A)

The number of α-type channel proteins (TC subclass 1.A) in the ten *Lactobacillus* spp. range from 10 to 14 per organism, with Lga having 10. Two of the PAP strains (Lpa, Lpl) and one of the OP strains (Lru) have the highest number (14 each) of channel proteins among the ten strains. Only the probiotic Lru strain has the two required homologues of TC# 1.A.30.1.3 necessary to form a flagellar motor (proton motive force (*pmf*)-dependent), MotAB. These MotA and MotB proteins comprise the stator element of the flagellar motor complex and are required for rotation of the flagellar motor [31]. The presence of these homologs in Lru indicates its potential for flagellar motility [32]. Further analysis of the motility operon of Lru revealed the presence of the constituent genes for flagellar motility. These results are in agreement with [32]. Most of the motile *Lactobacillus* species belong to the *L. salivarius* clade with Lru being a member of this clade [32]. The remaining nine strains included in this study are not members of the *L. salivarius* clade and seem to lack genes for flagellar motility.

Eight of the ten strains (excluding Lru and Lga) have a homologue of the CorA Metal Ion Transporter (MIT) Family (TC# 1.A.35). Members of this family are involved in the uptake of divalent cations, primarily Mg^2+^, but also Cd^2+^, Co^2+^, Ni^2+^, and Zn^2+^ [33,34]. Interestingly, all ten strains (OP and PAP) have two homologs of each of camphor resistance proteins (TC# 1.A.43.1.1 and TC# 1.A.43.1.2). Both transporters prevent fluoride toxicity by reducing the cytoplasmic concentration of fluoride ions [35].

### 3.3. Pore-Forming Toxins (TC Subclass 1.C)

Pore-forming toxins (PFTs) are included in TC subclass 1.C. The range of toxins was 3–7 in all ten strains, with the two PAP strains, Lac and Lpl, having 7 each. The PFTs observed in the strains are given in Table 3. All ten strains (OP and PAP) examined have one homolog of TC# 1.C.113.1.1, a hemolysin of the Hly III family (TC# 1.C.113). This hemolysin has been shown to have strong pore-forming activity and causes cell lysis of mammalian cells [36,37]. Another hemolysin that seems to be present in all of the strains is TC# 1.C.82.1.1, a member of the Pore-forming Amphipathic Helical Peptide (HP2–20) Family. Members of this family exhibit broad spectrum antibacterial and antifungal activities by creating pores in the cell membranes of target bacteria and fungi; however, little or no lysis of mammalian cells was observed. Thus, the representation of this family in all ten strains is indicative of the probiotic potential of these lactobacilli. The OP Lru strain encodes a homologue (TC# 1.C.24.1.1) of the Pediocin family (TC# 1.C.24). This protein is a Class IIa bacteriocin Pediocin PA-1 precursor of 62 aas and has strong bactericidal activity against *Listeria monocytogenes* after proteolytic processing [38].

Among the PAP strains, various PFTs were identified. Only Lac and Lpl encode members of the Plantaricin JK Family (TC# 1.C.30), with Lac having a homolog of TC# 1.C.30.1.3, a two-component bacteriocin (thermophilin) of *Streptococcus thermophilus*. It consists of an antibacterial peptide (ThmA) and an enhancing factor ThmB and participates in autolysin maturation and cell surface biogenesis [39]. This bacteriocin is also considered to be pivotal in virulence expression. It has antilisterial activity but lacks the YGNGV-C motif, typical of *Listeria*-active peptides. Lpl seems to possess a two-component bacteriocin (Plantaricin J/K) (TC# 1.C.30.1.1) which has demonstrated antifungal activities against *Candida albicans* [40]. This bacteriocin causes membrane potential dissipation and loss of the *pmf* and cytoplasmic K^+^, followed by cell death, possibly due in part by the release of reactive oxygen species [41]. Similar functions of plantaricin EF (TC# 1.C.29.1.1) have been reported [42]; a homolog was found in Lpl. Interestingly, only Lpa encodes a homolog (TC# 1.C.83.1.2) of the Gasserin Family (TC# 1.C.83). The hit PFT in TCDB is a butyrovibriocin of *Butyrivibrium fibrisolvens* AR10 and has broad spectrum antibacterial activity against isolates of *Butyrivibrium* [43].

Most of the PFTs observed in all ten strains seem to be bacteriocins. These antimicrobial peptides assist the *Lactobacillus* strains by killing other microbes in their surroundings. However, the presence of certain hemolysins in all ten lactobacilli is interesting, specifically in the context of the six OP strains, thus indicative of yet unrecognized pathogenic potential.

### 3.4. Holins (TC Subclass 1.E)

TC subclass 1.E consists of holins, which play numerous roles in bacterial cells such as release of toxins, cell lysis and death, biofilm formation, virulence and as antimicrobials to influence the transport of proteins to the extracellular environment [44]. These proteins can also be connected with the probiotic potential of Gram-positive bacteria such lactobacilli [44]. With regards to holins, the two PAP strains, Lpa and Lpl, contain the highest numbers (6 and 8, respectively) of such proteins. All ten strains have homologs of the putative 3–4 TMS Transglycosylase-associated Holin (T-A Hol) Family (TC# 1.E.43); functions of the members of this family are still not well understood. Three of the strains, Lbr, Lpl and Lre, have members of the CidA/LrgA Holin (CidA/LrgA Holin) Family, TC# 1.E.14. Members of this family affect antibiotic tolerance, survival during stationary phase, biofilm formation, and oxidative stress [44]. Their syntheses are regulated in a fashion that is different from that found in other bacterial species like *Staphylococcus aureus*, where a 2-component regulatory system, LytSR regulates these genes [45,46,47].

Initially, the roles of holins in programmed cell death in bacteria were debatable; however, recent findings suggest that these phage-encoded proteins may initiate lysis of the bacterial cell via murein hydrolase activity [48]. In our analysis we found murein hydrolase genes adjacent to the holin genes in most of the lactobacilli, showing that these holins may modulate the cell death process in these strains.

### 3.5. Bacterial Micro/NanoCompartment Shell Protein Pores (1.S)

TC subclass 1.S consists of bacterial microcompartment shell/pore-forming proteins (BMC-SP). In TCDB this subclass has two families; TC# 1.S.1 (BMC-SP1) and TC# 1.S.2 (BMC-SP2). Hexameric proteins known as microcompartment shell proteins form a tightly packed layer to constitute bacterial microcompartments (BMCs). These proteins tend to assemble into cyclic hexamers and have narrow central pores with diffusive molecular transport taking place via these pores [49]. Two of the lactobacilli, Lbr and Lre, have homologs of TC subclass 1.S. Both strains seem to have the propanediol use protein PduA, similar to that of the *Salmonella typhimurium* LT2 homolog (TC# 1.S.1.1.1). This latter protein is important for the degradation of 1,2-propanediol and has a hexameric structure, with each subunit of 99 aas, containing a well-defined pore [50]. The other BMC-SP identified in both strains is the PduB shell protein (TC# 1.S.2.1.2) of 270 aas of a propanediol use polyhedral body.

### 3.6. Secondary Carriers (TC Subfamily 2.A)

The number of secondary carriers in the ten strains range from 51–130, with Lbu having the least and Lpa having the highest number of these transport proteins. The major facilitator superfamily (MFS) (TC# 2.A.1) is well represented across the species with numbers ranging from 16 to 46. Three strains (Lac, Lbu and Lru) lack members of the Sugar Porter Family (TC# 2.A.1.1). Members of the Drug:H^+^ Antiporter-1 (12 Spanner) (DHA1) Family (TC# 2.A.1.2) and the Drug:H^+^ Antiporter-2 (14 Spanner) (DHA2) Family (TC# 2.A.1.3) were identified in all ten species. Interestingly, all of the ten lactobacilli have a multidrug resistance (MDR) efflux pump (TC# 2.A.1.2.20); this drug exporter confers resistance to fluoroquinolone and many other drugs [51]. With the exception of Lbu, Lre and Lru, the rest of the stains have a homolog of TC# 2.A.1.3.33, another MDR porter. Interestingly, the OP Lbr strain encodes a homolog of TC# 2.A.1.21.1, a macrolide efflux pump of *Streptococcus pyogenes*. This protein confers resistance to various macrolides including erythromycin, oleandomycin, azithromycin, and telithromycin [52,53]. Members of the Uncharacterized Major Facilitator-5 (UMF5) Family (TC# 2.A.1.46) are absent in three (Lbr. Lpa, Lpl) of the ten strains. On the basis of sequence similarity, members of this family are likely to be MDR pumps. The putative quinolone resistance protein (TC# 2.A.1.46.5) seems to be present in the same seven strains.

Only Lga seems to lack members of the Glycoside-Pentoside-Hexuronide (GPH): Cation Symporter Family (TC# 2.A.2). Proteins of this family catalyze the uptake of sugars. The Amino Acid-Polyamine-Organocation (APC) Superfamily (TC# 2.A.3) is the second largest family of secondary carriers after the MFS and is also well represented in the ten lactobacilli. Surprisingly, Lbu, an OP strain, seems to encode only four members of the APC family, while the remaining nine strains have 10–20 such proteins. These proteins catalyze the uptake of amino acids and their derivatives [54]. The presence of these systems in the strains gives them an advantage as external amino acids can be used directly for protein synthesis. In addition, these transporters may also be used for catabolism, resulting in the availability of energy, carbon and nitrogen which may be used by these bacterial species in various physiological processes [55]. Initially, the deficiency of transporters of the APC superfamily may point towards a lack of competitiveness for acquisition of nutrients; however, the presence of amino acid uptake porters in other families (e.g., secondary and primary active transporters such as ABC-type uptake porters (TC# 3.A.1) may compensate for this deficiency.

Members of the Cation Diffusion Facilitator (CDF) Family (TC# 2.A.4) seem to be present in all of the strains except Lpa. These secondary carriers primarily catalyze the efflux of heavy metals from cells and may also take them up into intracellular vesicles and organelles [56]. There are disparate patterns for proteins of the Resistance-Nodulation-Cell Division (RND) Superfamily (TC# 2.A.6) with Lac and Lpl (two PAP strains) lacking members, while the remaining strains encode only 1–2 proteins of the family. All of the strains have homologs of the Drug/Metabolite Transporter (DMT) Superfamily (TC# 2.A.7), with Lpl having the most (10) such proteins. All known members of this family transport small metabolites and drugs, either into or out of cells [57]. Three of the PAP strains, Lpa, Lpl and Lrh, each have a member of the 4 TMS Small Multidrug Resistance (SMR) Family (TC# 2.A.7.1). This protein (TC# 2.A.7.1.4) is an efflux pump of *E. coli* for quaternary ammonium compounds. This transport protein could prove to be useful for cell survival in the three PAP strains, as quaternary ammonium compounds have antibacterial function which they exert by damaging the cell membrane, resulting in the leakage of cell components and eventual cell death [58,59].

All of the strains have members of the Multidrug/Oligosaccharidyl-lipid/Polysaccharide (MOP) Flippase Superfamily (TC# 2.A.66) with a range of 2–7. Proteins of this family use cation (usually Na+) antiport to catalyze the efflux of their substrates [60]. Three of the PAP strains, Lpa, Lpl and Lrh, each has a homolog of TC# 2.A.66.1.33, which is an MDR pump for quinolones, indicative of resistance of these pathogens to quinolones, specifically, moxifloxacin, ciprofloxacin, and levofloxacin [61]. Among the OP strains, Lga seems to lack members of the Multi Antimicrobial Extrusion (MATE) Family (TC# 2.A.66.1). On the other hand, two OP strains, Lbr and Lbu, have an MDR pump (TC# 2.A.66.1.13); this protein exports fluroquinolones, biocides and tigecycline [62,63,64].

Other families that are represented in the strains examined here include the Cadmium Resistance (CadD) Family and the Threonine/Serine Exporter (ThrE) Family (TC# 2.A.77 and TC# 2.A.79, respectively). Additionally, members of the Novobiocin Exporter (NbcE) Family (TC# 2.A.115) are present in nine of the ten strains. Only Lru lacks proteins of this family, suggesting sensitivity of the strain to novobiocin.

### 3.7. Primary Active Transporters (TC Subclass 3.A)

The ATP-binding Cassette (ABC) Superfamily (TC# 3.A.1) is well represented across the ten lactobacilli. Prokaryotes and chloroplasts have ABC uptake systems, but these are absent in other compartments of the eukaryotic cell which have only ABC efflux systems. The pattern of distribution of these proteins in the ten lactobacilli is variable between the PAP and OP strains. However, the PAP strains generally have higher numbers of ABC transporters, with Lpa having the most with 160 such proteins, Lrh with 136, Lpl with 127 and Lac with 103. In the OP strains, Lcr has the most with 110, Lbu with 102, Lru with 93, Lbr with 86, Lga with 82 and Lre with only 62. The presence of large numbers of proteins found in this subclass point to efficient efflux and uptake capabilities of the ten strains, as they tend to be high affinity, but low efficiency transporters compared to secondary carriers. Clearly these “scavenger”-type systems play major roles for the metabolism of the ten lactobacilli. Their prevalence, and those of PTS transporters (see below) are consistent with a primarily fermentative mechanism for generating energy [65,66].

The Carbohydrate Uptake Transporter-1 (CUT1) Family (TC# 3.A.1.1) is well represented across the strains, with Lpa having the most, with 85 proteins. Members of the Carbohydrate Uptake Transporter-2 (CUT2) Family (TC# 3.A.1.2) are also present among the ten strains. Non-digestible oligosaccharides (NDOs) like raffinose, stachyose and fructo-oligosaccharides are resistant to enzymatic digestion by intestinal mucosal cells and are degraded by select bacterial species that possess the metabolic repertoire to degrade them. The NDOs mentioned above have been termed “prebiotics” and stimulate the growth of beneficial bacteria in the gut [67]. In our analysis, both Lac and Lcr have three components of TC# 3.A.1.1.28, a four component raffinose/stachyose transporter. The presence of homologs of such a transport system in the two strains suggests that these lactobacilli have the metabolic machinery to use both NDOs, which gives them a competitive advantage over their bacterial neighbors in colonizing the gut. In addition, both Lac and Lcr have complete systems of TC# 3.A.1.1.20, a fructo-oligosaccharide uptake porter, thus, providing both strains an edge in using another NDO. The remaining eight strains also have considerable numbers of proteins from both families (TC# 3.A.1.1 and TC # 3.A.1.2), showing that these lactobacilli are adept at taking sugars up from their surroundings.

Transporters for the uptake of polar amino acids (TC # 3.A.1.3) are present in all ten strains with a range of 13–26 proteins. However, members of the Hydrophobic Amino Acid Uptake Transporter (HAAT) Family (TC# 3.A.1.4) are present only in three PAP strains, Lpa, Lpl and Lrh, and one OP strain, Lru. These four strains contain homologs of all five components of a branched chain hydrophobic amino acid transporter (TC# 3.A.1.4.10). This transporter has been shown to be important for the virulence of *Streptococcus pneumoniae* [68]. Similarly, the presence of this branched chain amino acid transporter in the PAP strains could assist these pathogens in causing disease. With the exception of Lre, all of the lactobacilli have protein of the Peptide/Opine/Nickel Uptake Transporter (PepT) Family (TC # 3.A.1.5).

Each of the ten strains examined seem to have complete systems of the Polyamine (putrescine/spermidine) uptake porter (TC# 3.A.1.11.7) of the Polyamine/Opine/Phosphonate Uptake Transporter (POPT) Family (TC# 3.A.1.11). These polyamines serve various physiological and pathological roles in bacterial species, such as cell growth, acid resistance, biofilm formation, and virulence [69].

Prokaryotic ABC efflux systems (TC# 3.A.1.100–3.A.1.199), have similar patterns in all ten strains. The substrates of these transport proteins include drugs, antimicrobial peptides, proteins, and amino acids. Lpa has the largest number of these ABC efflux systems with 55 proteins.

All of the lactobacilli possess F-type ATPases (TC# 3.A.2) for energy interconversion. An important feature of these systems is the reversibility of the enzyme complex for either the establishment of a proton motive force at the expense of ATP, or for ATP synthesis at the expense of the *pmf* [70]. Members of the P-type ATPase (P-ATPase) Superfamily (TC# 3.A.3) are present in all ten strains, ranging from 3 to 9 per organism and have different substrate specificities. The general secretory pathway (Sec) (TC# 3.A.5) seems to functional with components in all ten strains.

### 3.8. Decarboxylation-Driven Transporters (TC Subclass 3.B)

Transport proteins of TC# 3.B are present in Lbu, Lcr, Lpa and Lrh. All four strains have at least one Na^+^-transporting carboxylic acid decarboxylase family member (TC# 3.B.1). This protein couples decarboxylation to the extrusion of sodium ions [71].

### 3.9. Oxidoreduction Driven Transporters (TC Subclass 3.D)

*Lactobacilli* include anaerobic and aero-tolerant species and are classically regarded as strictly fermentative [72]. However, some species show aerobic respiration via a rather simple and non-redundant electron transport chain consisting of an NADH dehydrogenase, a menaquinol pool and a *bd*-type cytochrome. Moreover, proton pumping electron transfer complexes are characteristic of aerobic bacteria species; however, constituents seem to be present in the lactobacilli. These include members of the H^+^ or Na^+^-translocating NADH Dehydrogenase (NDH) Family (TC# 3.D.1) and the Proton-translocating Cytochrome Oxidase (COX) Superfamily (TC# 3.D.4).

### 3.10. Possible Group Translocators (TC Class 4)

All ten strains have homologs of phosphotransfer-driven group translocators (PTS) (TC# 4.A). The PAP strains have far more of these proteins (range, 23–64) compared to the OP strains (range, 3–31). The presence of these translocators in higher numbers in the PAP strains suggests that much of the sugar transport mediated by these lactobacilli, particularly in Lpa (61 proteins) and Lrh (54 proteins) is mediated by PTS transporters. Our results for Lac (23 proteins) and Lpl (44) are also in agreement with [73] and [20], suggesting that PTS sugar uptake systems are the major transport systems in these two species. Another important aspect is the lack of members of the PTS Mannose-Fructose-Sorbose (Man) Family (TC# 4.A.6) in Lre; this is in accordance with [74], in which similar observations were reported. The remaining nine strains have homologs of this family. Members of TC# 4.A.6 usually exhibit broad specificity for a range of sugars, rather than being specific for just one or a few sugars.

Four strains, Lbr, Lcr, Lga, and Lpa, have members of the Proposed Fatty Acid Group Translocation (FAT) family (TC# 4.C.1) in TC subclass 4.C of Acyl CoA Ligase-coupled transporters. While the activities of these ligases have been established [75], their association with transport is still not accepted [76]. Each of the *Lactobacillus* strains has 1–4 polysaccharide synthase/exporters (TC# 4.D). These proteins have exopolysaccharide synthesis activities that are considered to be coupled to the secretion of polysaccharides [77]. As exopolysaccharides are known to play pivotal roles in biofilm formation, they may have similar functions in the ten strains. Also, six strains (Lbr, Lpa, Lpl, Lre, Lrh, and Lru) have one protein each of the lysylphosphatidylglycerol synthase/flippases (TC# 4.H). Proteins belonging to this subclass confer resistance to cationic antimicrobial agents such as daptomycin [78].

### 3.11. Transmembrane Electron Carriers (TC Class 5)

Members of this class are associated with cellular energetics. Of the ten strains, only Lpl has members of this subclass. It has two components of an anaerobic, respiratory, membrane-bound nitrate reductase (TC# 5.A.3.1.1) and one component of another nitrate reductase (TC# 5.A.3.1.2). This suggests that Lpl may have the capacity to use nitrate as an electron acceptor during anaerobic growth. These observations are in agreement with previous research, which revealed that *narGHJI* genes, encoding a nitrate-reductase A complex, are present and expressed in Lpl. This species is able to reduce nitrate to nitrite only in the presence of both heme and menaquinone [79]. With the exception of Lac, all lactobacilli have proteins of the transmembrane 1-electron transfer carrier subclass (TC# 5.B). Three PAP strains, Lpa, Lpl and Lrh, have six components each of the eight component flavin-based extracellular electron transfer chain TC# 5.B.14.1.1. Similar to this transfer chain, characterized in *Listeria monocytogenes*, the lactobacilli may use the same type of flavin shuttle for transmembrane electron transfer.

### 3.12. Auxiliary Transporters (TC Subclass 8.A)

Eight of the ten lactobacilli, all except Lbr and Lre, have proteins of the Cytoplasmic Membrane-Periplasmic Auxiliary-1 (MPA1) Protein with Cytoplasmic (C) Domain (MPA1-C or MPA1+C) Family (TC# 8.A.3).It has been proposed that Members of this family function in combination with the Polysaccharide Transport Family (TC# 2.A.66.2). Homologs of the Stomatin/Podocin/Band 7/Nephrosis.2/SPFH (Stomatin) Family (TC# 8.A.21) are also present in the strains of lactobacilli; these homologs probably function in membrane stress adaptation [80].

### 3.13. Incompletely Characterized Transport Systems (TC Class 9)

**Figure 1:**
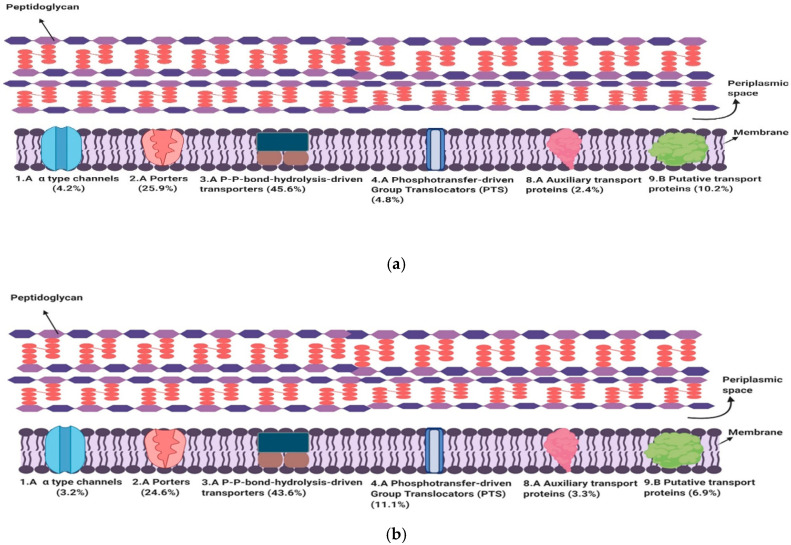
The average percentages of major classes in the six only probiotic strains (**a**) and the four pathogenic and probiotic strains (**b**). (**a**) The best represented TC subclasses for transport proteins in the six OP strains are primary active transport proteins (3.A) (45.6%) followed by secondary carriers (2.A) (25.9%). Out of the major classes, the auxiliary transport proteins are the least well represented within the OP strains (2.4%). (**b**) In the PAP strains, TC subclass 3.A is also the best represented class followed by 2.A. However, in these strains, the α-type channels (2.A) are the least well represented.

A few (1–5 per organism) poorly characterized transporters are encoded within the genomes of the ten strains. The overall range of proteins in this subclass for all ten strains is 17–34. An overview of the average percentages of the various TC classes observed in the OP and PAP strains is presented in Figure 1. 

### 3.14. Differences in Substrates Transported by PAP and OP Strains

The substrate specificities of the transport proteins of all ten strains (PAP and OP) are shown in Table 4. Among the four PAP lactobacilli, Lac seems to have the smallest number of transport proteins with unknown function (73), whereas the range among the other three strains is 95–131. These numbers are much higher than the OP strains which have 66–95 proteins with unknown substrate specificities. Correspondingly, the PAP strains have more peptide and protein transporters (30–45) in comparison to the OP strains (19–36). Most of these transporters seem to bebacteriocins, and are thus indicative of the probiotic potential of all of the strains. On average, the PAP strains have more cation transporters than the OP strains (30–54 versus 25–43). This shows that these lactobacilli are more adept in resistance to heavy metals, ionic homeostasis, and osmotic regulation. In all ten strains, the numbers of anion transporters are significantly less than those of the cationic porters with a range of 4–9. The three main anions transported are chloride, fluoride, and phosphate.

The numbers of amino acid transporters in the PAP strains are higher than of the OP strains (36–47 vs. 30–39). Amino acid transporters have been shown to play multiple roles in bacterial pathogens and are pivotal to their growth and persistence in their ecological niches. These transporters are also associated with increased virulence of the species that encode them [81,82]. The PAP strains have more drug exporters (12–25) than their probiotic counterparts (8–21). Lpl has the most (25). Thus, it may indicate increased antibiotic resistance in the strain; however, this may not be necessarily true, as drug efflux pumps may have numerous specificities.

The most commonly exported drugs by the ten lactobacilli include fluoroquinolones, macrolides, aminoglycosides, and bacitracin.

Various transporters for vitamins are present in the lactobacilli; however, the PAP strains have somewhat more of these transporters (7–16 vs. 7–13). Most of the vitamins transported belong to the B vitamin family and include riboflavin, thiamine, folic acid, and biotin. Thiamine is an essential component required by bacterial species for glycolysis, branched chain amino acid metabolism, and nucleotide metabolism [83]. All of the strains have components of a putative thiamine porter (TC# 3.A.1.30.2), thus showing the potential of these strains to take up thiamine from their respective environments. The PAP strains also have higher numbers of sugar transporters than the OP strains (30–61 versus 8–43). The major sugars transported by the ten strains include maltose, trehalose, rhamnose, glucose, fructose, lactose and raffinose. 

### 3.15. Major Families/Superfamilies Found in Lactobacillus Strains

The four best represented families in all of the ten strains are shown in Table 5. Members of the ATP-binding Cassette (ABC) Superfamily (TC# 3.A.1) are abundant in both the PAP and OP strains with a range of 62–160. Overall, a total of 1061 proteins of this family are present in the lactobacilli, and average percentages of these proteins relative to the total transport proteins is 22–41.4%. Interestingly, the remaining three prominent families (the Major Facilitator Superfamily (MFS) (TC#2.A.1), the Amino Acid-Polyamine-Organocation (APC) Superfamily (TC#2.A.3) and the Drug/Metabolite Transporter (DMT) Superfamily (TC#2.A.7) have a much smaller average percentage (1.3–6.1%) as compared to the ABC transport proteins. This is to be expected for fermentative microbes with few *pmf*-generating electron flow carriers [66].

## 4. Discussion

The genus *Lactobacillus* is regarded as the largest group of the lactic acid bacterial (LAB) and consists of many species that are associated with fermentation of plants, meat, and milk [84]. Members are also inhabitants of the mammalian gut, though various species may also thrive in other anatomical locations like the oral cavity and the urinary and genital tracts [85]. As these microbes have a broad range of environmental niches, they are equipped with the necessary metabolic repertoire (transporters, enzyme systems) to cope with fluctuations in nutrient availability. Lactobacilli are routinely used as probiotics due to their health benefits; however, some strains have the potential to cause serious clinical infections such as bacteremia, endocarditis, and intra-abdominal abscesses [86].

The present transportome analysis provides interesting facts about the mechanistic, physiological, metabolic, and pathogenic capabilities of the ten strains. All of the lactobacilli in this study encode proteins that enable them to thrive in individual ecological niches. The metabolites transported by the strains seem to assist them in their extracellular probiotic lifestyles. For, instance all ten lactobacilli have numerous vitamin and sugar transporters that help in the use of available metabolites, and in turn, these probiotic species can convert them into compounds that may be of benefit to the host. Some of the ABC transporters observed in the ten strains are involved in the export of antimicrobial peptides (e.g., lantibiotics, bacteriocins and competence peptides) in agreement with the suggestions of de Jong et al., 2006 [87]. Our results show that antimicrobial peptide export via ABC transporters is common in lactobacilli and probably contributes to their probiotic potential. Overall, the large contingents of ABC transporters can also be associated with carbohydrate metabolic flexibility [84].

All ten strains examined have homologs of pore-forming toxins (PFTs), although most of these toxins, prevalent in the lactobacilli, are bacteriocins. Many of the toxins identified in the strains have antifungal properties; the antifungal effects of lactobacilli have been studied in some detail [88,89,90]. Some of the toxic proteins, however, have pathogenic effects on mammalian cells. In the case of the PAP strains, these toxins may assist in causing disease symptoms, and for the OP strains, the presence of these toxins may indicate yet unrecognized pathogenic potential. The PFTs may cause pathogenesis directly, when the lactobacilli strains translocate to extra-abdominal locations in the body.

The presence of holins in the genus *Lactobacillus* has been reported previously [91]. Although the identification of holins is hindered by their small sizes and often, low degrees of sequence similarity, they do share overall structural and functional features that are commonly conserved in prokaryotes and bacteriophages. The presence of holin homologs in all ten lactobacilli suggests that these proteins contribute to the probiotic and possibly the pathogenic attributes of both PAP and OP strains.

Lactobacilli can use a variety of carbohydrates which reflects the nutrients available in their respective environments. It has been suggested that PTS transporters are the main carbohydrate transporters in *Lactobacillus* strains [92]. This suggestion was confirmed through genomic studies, especially for Lac, Lga and Lpl, and results correlated with the predicted broad carbohydrate use capabilities for these strains. Our results are in agreement with previous research, as eight strains contain large numbers of these transporters. Moreover, the higher numbers of PTS transporters in the PAP strains is interesting. It has been suggested that several of the PTS transporters may be linked to virulence; however, this depends upon the types of bacterial species and hosts involved. This is certainly an area of study that deserves more attention.

A comparative analysis of the transport proteins of the ten lactobacilli described here with strains of *E. coli* (extracellular and intracellular pathogens, probiotics, and commensals), intracellular pathogens of *Salmonella* and seven extracellular *Bacteroides* strains (both pathogenic and probiotic) provides interesting comparisons. The percentages of α-type channels in strains of the previous studies were 3.4 to 4.2% for *E. coli* and *Salmonella* strains and 2.9 to 5.4% for the *Bacteroides* strains, and the percentages of these channel proteins in the lactobacilli are comparable, 2.7 to 4.8%.

As Gram-positive firmicutes, the strains in this study lack β-barrel porins altogether; not one of these proteins was found to be encoded within the genomes of the ten strains. PFTs accounted for 0.2–1.4% in *E. coli* and *Salmonella* and 0.3–0.9% for the *Bacteroides* strains, but the overall percentages in the lactobacilli proved to be significantly higher (0.7–2.3%). However, similar to *E. coli* and *Salmonella,* there are no members of the Membrane Attack Complex/Perforin Family (MACPF) [93] in the lactobacilli, and the larger percentages of PFTs is primarily due to bacterium-targeting bacteriocins. Additionally, the percent of holins in the *Lactobacillus* strains is 0–1.9%, quite similar to the percentage of holins in *E. coli* and *Salmonella* (0.8–1.3%) and *Bacteroides* (0.5–0.8%).

The overall percent range of secondary porters (subclass 2.A) in the lactobacilli is 20.4–35.6%; this is similar to and overlapping with the *Bacteroides* strains (20–26.6%) and the *E. coli* and *Salmonella* strains (27.4–32.5). However, much higher percentages (35.9–55.7%) of the primary pyrophosphate hydrolysis-driven transporters (TC subclass 3.A) are present in the *Lactobacillus* strains; in the case of two probiotic strains (Lbu and Lru), these transport proteins comprise over 50% of the overall transportome! The percentages of these transporters were much lower in *E. coli*/*Salmonella* (26.9–32.5%) and *Bacteroides* (20.3–26.6%). Considering that all four genera are inhabitants of the mammalian gut, the higher numbers of primary pyrophosphate hydrolysis driven transporters in the lactobacilli may give them metabolic advantages over the other strains for nutrient acquisition and colonization in shared, largely anaerobic, ecological niches. This would be first, because the primary active transporters and PTS systems generally exhibit higher affinities for their substrates than secondary carriers, and second, because only the lactobacilli generate ATP primarily via substrate level phosphorylation [65,83].

The percentages of oxidoreduction-driven transporters are much lower (0–1.7%) in the primarily fermentative lactobacilli by comparison with the heterotrophic and more versatile *E. coli*/*Salmonella* (4.1–5.9%) and *Bacteroides* (4.7–6.7%) species. This is as expected for the lactobacilli as noted above, although some of these strains may perform a limited amount of aerobic respiration via rather simple electron transport chains. Additionally, the lactobacilli contain more auxiliary transport proteins (1.8–3.9%) than *E. coli* and *Salmonella* (1.7–2.6), but far fewer than *Bacteroides* (12–23%). However, the *Lactobacillus* strains have much higher overall percentages (5.6–11.4%) of recognized transporters of unknown biochemical mechanism than the strains (*E. coli*/*Salmonella,* 0.8–1.2% and *Bacteroides,* 0.3–1.1%) of our previous studies. Overall, we assume that the transportomes of the strains included in the present communication and previous studies are finely tuned according to their lifestyles (intra-organismal and free living, extra- and intra-cellular, probiotic, and pathogenic) requirements.

With respect to the genome size of the ten strains in this study (see Table 1), the larger sizes of three of the PAP strainsLpa, Lpl, and Lrh may explain patterns of results observed in this study. For instance, these three strains have much higher number of drug transporters, amino acid transporters, sugar transporters and unknown transporters in comparison to the other strains. The larger genome sizes of the three PAP strains equips them with higher numbers of transport proteins (range of 407–458) in comparison to the remaining seven strains (range of 246–328). Due to larger transportomes, the three strains are able to flex their metabolic muscle better than the other strains, thus enabling them to be major players in acquiring different nutrients in their respective ecological niches.

In this study, the transport proteins comprise a range of 14.5–17.9% of the proteomes of the ten strains examined, substantially higher than the percentages noted for the Gram-negative bacteria of our earlier studies. The overall transportomes of the lactobacilli reflect key features of their metabolic capabilities, pathogenic and probiotic potential, and reduced genome sizes, which are directly responsible for their reduced biosynthetic capacities. Additionally, the presence of PFTs and higher proportions of drug exporters in both OP and PAP strains is interesting as this may point toward yet undisclosed hidden pathogenic characteristics. Future analysis of the observed drug transporters would be intriguing, as it may enhance our understanding of patterns of antibiotic resistances in *Lactobacillus* spp. and will also be helpful in devising effective treatments for antimicrobial resistance among these strains. Additionally, the high numbers of carbohydrate and amino acid transporters are undoubtedly important for niche adaption. As this genus has a broad range of environmental niches, the transportomes of these species enable them to cope with and benefit from the differing nutrients they encounter, particularly oligosaccharides, peptides, and glycopeptides. Lactobacilli are well-known for their antibacterial effects by producing and exporting various types of bacteriocins. We also found a wide range of bacteriocins in the ten strains, thus confirming some of their probiotic attributes; these antimicrobial peptides could be further studied for the improvement of the probiotic potential of the strains and for the development of novel types of antibiotic and antifungal agents, all through the use of genetic manipulation techniques. Further efforts would also enable the identification of potential diseases that may be caused by the OP *Lactobacillus* strains.

The mammalian gut has a plethora of microbial residents that are in constant contact with their host and stuck in a constant battle for nutrient acquisition and niche colonization with their microbial counterparts. The transportome of these microbial residents is pivotal for their survival and persistence in the gut. Results from this communication in conjunction with our previous reports on other residents of the mammalian gut may provide valuable insights about the transportome of the mammalian gut bacteriome. Further comparative genomic studies on other gut residents will also be of value and comparisons could be made to increase our current understandings.

To reiterate and summarize, as of now, the OP strains included in this study have no recorded pathogenic effects. Based on results presented in this study; however, the transporters identified in these strains will help in the generation of modified lactobacilli strains with increased probiotic and decreased pathogenic potential. We hope this study will also be of value for the development of potential therapeutics for the pathogenic lactobacilli in the future.

## Figures and Tables

**Table 1:** Overview of the ten *Lactobacillus* species included in this study.

Strain	Abbreviation	Accession #	Genome Size (Mbp)	Total # of Proteins Identified	Transport Proteins (% of Total)	Location in Host	Relationship with Host
*Lactobacillus acidophilus NCFM*	Lac	NC_006814.3	1.99	303	17.1	GIT, oral cavity, vagina	Probiotic; may cause endocarditis
*Lactobacillus brevis ATCC 367*	Lbr	NC_008497.1	2.29	327	15.4	GIT, vagina	Probiotic; non-pathogenic
*Lactobacillus delbrueckii subsp. bulgaricus ATCC 11842*	Lbu	NC_008054.1	1.87	246	15.7	GIT	Probiotic; non-pathogenic
*Lactobacillus crispatus ST1*	Lcr	NC_014106.1	2.04	328	17.8	GIT, vagina	Probiotic; non-pathogenic
*Lactobacillus gasseri ATCC*	Lga	NC_008530.1	1.89	286	16.3	GIT, vagina	Probiotic; non-pathogenic
*Lactobacillus paracasei JCM 8130*	Lpa	NZ_AP012541.1	3.00	458	16.6	GIT	Probiotic and pathogenic
*Lactobacillus plantarum WCFS1*	Lpl	NC_004567.2	3.31	434	14.5	GIT	Probiotic and pathogenic
*Lactobacillus reuteri DSM 20016*	Lre	NC_009513.1	2.00	281	14.9	GIT, urinary tract, skin	Probiotic; non-pathogenic
*Lactobacillus rhamnosus GG*	Lrh	NC_013198.1	3.01	407	14.7	GIT, vagina	Probiotic and pathogenic
*Lactobacillus ruminis ATCC 27782*	Lru	NC_015975.1	2.07	289	15.8	GIT	Probiotic; non-pathogenic

GIT- Gastrointestinal Tract.

**Table 2:** Overview of *Lactobacillus* transport protein numbers (left) and percentages (right), based on TC subclass. PAP strains are in bold.

**TC Subclass and Description**	**Lac**	Lbr	Lbu	Lcr	Lga	**Lpa**	**Lpl**	Lre	**Lrh**	Lru	**Lac**	Lbr	Lbu	Lcr	Lga	**Lpa**	**Lpl**	Lre	**Lrh**	Lru
1.A, α-type channels	**12**	13	11	13	10	**14**	**14**	13	**11**	14	**3.9**	4.0	4.5	4.0	3.5	**3.1**	**3.2**	4.6	**2.7**	4.8
1.B, β-barrel porins	**0**	0	0	0	0	**0**	**0**	0	**0**	0	**0**	0	0	0	0	**0**	**0**	0	**0**	0
1.C, Pore-forming toxins	**7**	3	4	5	4	**4**	**7**	3	**3**	4	**2.3**	0.9	1.6	1.5	1.4	**0.9**	**1.6**	1.1	**0.7**	1.4
1.E, Holins	**1**	5	0	3	3	**6**	**8**	4	**5**	3	**0.3**	1.5	0	0.9	1.1	**1.3**	**1.9**	1.4	**1.2**	1.0
1.S: Bacterial Micro/Nano Compartment Shell Protein Pores	**0**	3	0	0	0	**0**	**0**	3	**0**	0	**0**	0.9	0	0	0	**0**	**0**	1.1	**0**	0
2.A, Porters (uniporters, symporters, antiporters)	**79**	114	51	68	66	**94**	**130**	100	**89**	59	**26.0**	34.8	20.8	20.7	23.1	**20.5**	**30.0**	35.6	**21.9**	20.4
3.A, P-P-bond-hydrolysis-driven transporters	**149**	128	137	161	123	**201**	**170**	101	**172**	148	**49.2**	39.1	55.7	49.1	43.0	**43.8**	**39.2**	35.9	**42.3**	51.0
3.B, Decarboxylation-driven transporters	**0**	0	1	2	0	**3**	**0**	0	**3**	0	**0**	0	0.4	0.6	0	**0.7**	**0**	0	**0.7**	0
3.D, Oxidoreduction-driven transporters	**2**	3	0	4	4	**3**	**3**	5	**0**	3	**0.7**	0.9	0	1.2	1.4	**0.7**	**0.7**	1.7	**0**	1.0
4.A Phosphotransfer-driven Group Translocators (PTS)	**23**	3	7	30	31	**61**	**44**	2	**54**	13	**7.6**	0.9	2.8	9.2	10.8	**13.3**	**10.1**	0.7	**13.3**	4.5
4.B, Nicotinamide ribonucleoside uptake transporters	**0**	1	0	0	0	**0**	**0**	0	**0**	0	**0**	0.3	0	0	0	**0**	**0**	0	**0**	0
4.C, Acyl-CoA ligase-coupled transporters	**0**	1	0	1	1	**1**	**0**	0	**0**	0,	**0**	0.3	0	0.3	0.35	**0.2**	**0**	0	**0**	0
4.D, Polysaccharide synthase exporters	**2**	4	1	2	2	**4**	**4**	1	**2**	3	**0.7**	1.3	0.4	0.6	0.65	**0.9**	**0.9**	0.4	**0.5**	1.0
4.F: Choline/Ethanolamine Phosphotransferase 1 (CEPT1)	**0**	1	1	1	1	**1**	**0**	1	**0**	1	**0**	0.3	0.4	0.3	0.3	**0.2**	**0**	0.4	**0**	0.4
4.H: Lysylphosphatidylglycerol Synthase/Flippases	**0**	1	0	0	0	**1**	**1**	1	**1**	1	**0**	0.3	0	0	0	**0.2**	**0.2**	0.4	**0.2**	0.4
5.A, Transmembrane two-electron transfer carriers	**0**	0	0	0	0	**0**	**3**	0	**0**	0	**0**	0	0	0	0	**0**	**0.7**	0	**0**	0
5.B: Transmembrane 1-electron transfer carriers	**0**	3	1	3	3	**11**	**8**	4	**12**	1	**0**	0.9	0.4	0.9	1.1	**2.4**	**1.9**	1.4	**3.0**	0.4
8.A, Auxiliary transport proteins	**9**	11	5	6	7	**17**	**11**	9	**16**	4	**3.0**	3.4	2.0	1.8	2.5	**3.7**	**2.5**	3.2	**3.9**	1.4
9.A, Recognized transporters of unknown biochemical mechanism	**2**	1	2	1	1	**3**	**4**	3	**5**	2	**0.7**	0.3	0.8	0.3	0.3	**0.7**	**0.9**	1.1	**1.2**	0.8
9.B, Putative transport proteins	**17**	32	25	28	30	**34**	**27**	31	**34**	33	**5.6**	9.8	10.2	8.5	10.5	**7.4**	**6.2**	11.0	**8.4**	11.4
Total	**303**	327	246	328	286	**458**	**434**	281	**407**	289	**100**	100	100	100	100	**100**	**100**	100	**100**	100

**Table 3:** Occurrence of pore-forming toxins in the ten *Lactobacillus* strains.

TCID	Family	Function	Lac	Lbr	Lbu	Lcr	Lga	Lpa	Lpl	Lre	Lrh	Lru	Type of Strains
1.C.24.1.1	Pediocin Family	Pore formation	0	0	0	0	0	0	0	0	0	1	OP
1.C.29.1.1	Plantaricin EF Family	Pore formation	0	0	0	0	0	0	1	0	0	0	PAP
1.C.30.1.1	Plantaricin JK Family	Pore formation	0	0	0	1	0	1	0	0	0	0	PAP and OP
1.C.30.1.3	Plantaricin JK Family	Pore formation	1	0	0	0	0	0	0	0	0	0	PAP
1.C.75.1.7	*Serratia*-type Pore-forming Toxin (S-PFT) Family	Pore formation	1	0	0	0	0	0	0	0	0	0	PAP
1.C.82.1.1	Pore-forming Amphipathic Helical Peptide (HP2-20) Family	Pore formation	1	1	1	1	1	1	1	1	1	1	PAP and OP
1.C.83.1.2	Gassericin Family	Pore formation	0	0	0	0	0	1	0	0	0	0	PAP
1.C.105.2.9	*Bacillus thuringiensis* Vegetative Insecticidal Protein-3 (Vip3) Family	Pore formation	1	0	0	1	1	0	0	0	0	0	PAP and OP
1.C.109.1.5	Bacterial Hemolysin A Family	Pore formation	1	1	1	1	1	1	1	1	1	1	PAP and OP
1.C.113.1.1	Hly III Family	Pore formation	1	1	1	1	1	1	1	1	1	1	PAP and OP
1.C.126.1.2	HlyC Family of Haemolysins	Pore formation	0	0	1	0	0	0	0	0	0	0	OP
1.C.126.1.3			0	0	0	1	0	0	0	0	0	0	OP

OP—only probiotic strains; PAP—both pathogenic and probiotic strains.

**Table 4:** Overview of predicted substrate specificities of transport proteins (expressed in numbers) in the ten *Lactobacillus* strains. The PAP strains are in bold.

**Substrate Category**	**Lac**	Lbr	Lbu	Lcr	Lga	**Lpa**	**Lpl**	Lre	**Lrh**	Lru
Inorganic Anions	**7**	8	3	6	4	**8**	**5**	6	**7**	9
Inorganic Cations	**30**	43	27	39	25	**38**	**54**	38	**31**	27
Amines	**4**	5	4	4	4	**4**	**5**	4	**4**	4
Amino acids	**36**	31	30	35	37	**43**	**47**	39	**38**	35
Non-selective	**44**	52	39	45	46	**75**	**77**	36	**57**	44
Drugs	**12**	21	13	14	16	**20**	**25**	8	**19**	11
Nucleobases, Nucleosides, Nucleotides	**26**	22	13	24	17	**14**	**19**	18	**13**	10
Proteins, Peptides	**30**	27	24	29	19	**45**	**35**	20	**37**	36
Sugars and Sugar Derivatives	**30**	8	13	43	33	**61**	**56**	12	**63**	25
Lipids	**4**	2	3	4	4	**3**	**2**	2	**3**	2
Vitamins	**7**	13	11	10	7	**16**	**14**	11	**15**	12
Unknown	**73**	95	66	75	74	**131**	**95**	87	**120**	74
Total	**303**	327	246	328	286	**458**	**434**	281	**407**	289

**Table 5:** Tabulation of the four largest transport protein families encoded within the genomes of the ten *Lactobacillus* strains. Both the total number of proteins (left) and average percentages of major family members (right) are shown. PAP strains are marked in bold.

Family name, abbreviation, and TC#	Lac	Lbr	Lbu	Lcr	Lga	Lpa	Lpl	Lrh	Lre	Lru	Total #	Lac	Lbr	Lbu	Lcr	Lga	Lpa	Lpl	Lrh	Lre	Lru	Average%
**The Major Facilitator Superfamily (MFS) (TC#2.A.1)**	**12**	32	10	13	15	**25**	**38**	**30**	23	11	209	**3.9**	9.8	4.1	3.9	5.2	**5.5**	**8.8**	**7.3**	8.2	3.8	6.1
**The Amino Acid-Polyamine-Organocation (APC) Superfamily (TC#2.A.3)**	**15**	12	4	15	13	**10**	**12**	**13**	20	6	120	**4.9**	3.6	1.6	4.6	4.5	**2.2**	**2.8**	**3.2**	7.1	2.1	3.7
**The Drug/Metabolite Transporter (DMT) Superfamily (TC#2.A.7)**	**5**	6	3	3	4	**4**	**10**	**4**	6	1	46	**1.7**	1.8	1.2	0.9	1.4	**0.8**	**2.3**	**1.0**	2.1	0.3	1.3
**The ATP-binding Cassette (ABC) Superfamily (TC#3.A.1)**	**103**	86	102	110	82	**160**	**127**	**136**	62	93	1061	**33.9**	26.2	41.4	33.5	28.7	**34.9**	**29.2**	**33.4**	22.0	32.1	31.6
**Totals**	**135**	136	119	141	114	**199**	**187**	**183**	111	111	1436/3359	**44.4**	41.4	48.3	42.9	39.8	**43.4**	**43.1**	**44.9**	39.4	38.3	42.7

## Supplementary Materials

The following are available online at https://www.mdpi.com/2073-4425/11/10/1234/s1, Table S1: Supplementary information about the ten *Lactobacillus* strains.

## References

[1] Walter J. (2008). Ecological role of lactobacilli in the gastrointestinal tract: Implications for fundamental and biomedical research. Appl. Environ. Microbiol..

[2] Campana R., van Hemert S., Baffone W. (2017). Strain-specific probiotic properties of lactic acid bacteria and their interference with human intestinal pathogens invasion. Gut Pathog..

[3] Rossi F., Amadoro C., Colavita G. (2019). Members of the Lactobacillus Genus Complex (LGC) as Opportunistic Pathogens: A Review. Microorganisms.

[4] Martín R., Miquel S., Ulmer J., Kechaou N., Langella P., Bermúdez-Humarán L.G. (2013). Role of commensal and probiotic bacteria in human health: A focus on inflammatory bowel disease. Microb. Cell Fact..

[5] Baron S. (1996). Medical Microbiology.

[6] Salvetti E., Torriani S., Felis G.E. (2012). The Genus Lactobacillus: A Taxonomic Update. Probiotics Antimicrob. Proteins.

[7] Sanchez S., Demain A.L. (2008). Metabolic regulation and overproduction of primary metabolites. Microb. Biotechnol..

[8] Wells J.M. (2011). Immunomodulatory mechanisms of lactobacilli. Microb. Cell Fact..

[9] Fijan S. (2014). Microorganisms with claimed probiotic properties: An overview of recent literature. Int. J. Environ. Res. Public Health.

[10] Chen C.C., Lai C.C., Huang H.L., Huang W.Y., Toh H.S., Weng T.C., Chuang Y.C., Lu Y.C., Tang H.J. (2019). Antimicrobial Activity of Lactobacillus Species against Carbapenem-Resistant Enterobacteriaceae. Front. Microbiol..

[11] Gaspar C., Donders G.G., Palmeira-de-Oliveira R., Queiroz J.A., Tomaz C., Martinez-de-Oliveira J., Palmeira-de-Oliveira A. (2018). Bacteriocin production of the probiotic Lactobacillus acidophilus KS400. AMB Express.

[12] Azad M.A.K., Sarker M., Li T., Yin J. (2018). Probiotic Species in the Modulation of Gut Microbiota: An Overview. Biomed Res. Int..

[13] Ding Y.H., Qian L.Y., Pang J., Lin J.Y., Xu Q., Wang L.H., Huang D.S., Zou H. (2017). The regulation of immune cells by Lactobacilli: A potential therapeutic target for anti-atherosclerosis therapy. Oncotarget.

[14] Yan F., Polk D.B. (2010). Probiotics: Progress toward novel therapies for intestinal diseases. Curr. Opin. Gastroenterol..

[15] Cannon J.P., Lee T.A., Bolanos J.T., Danziger L.H. (2005). Pathogenic relevance of Lactobacillus: A retrospective review of over 200 cases. Eur. J. Clin. Microbiol. Infect. Dis..

[16] Harty D.W., Oakey H.J., Patrikakis M., Hume E.B., Knox K.W. (1994). Pathogenic potential of lactobacilli. Int. J. Food Microbiol..

[17] Tang F., Saier M.H. (2014). Transport proteins promoting Escherichia coli pathogenesis. Microb. Pathog..

[18] Do J., Zafar H., Saier M.H. (2017). Comparative genomics of transport proteins in probiotic and pathogenic Escherichia coli and Salmonella enterica strains. Microb. Pathog..

[19] Zafar H., Saier M.H. (2018). Comparative genomics of transport proteins in seven Bacteroides species. PLoS ONE.

[20] Altermann E., Russell W.M., Azcarate-Peril M.A., Barrangou R., Buck B.L., McAuliffe O., Souther N., Dobson A., Duong T., Callanan M. (2005). Complete genome sequence of the probiotic lactic acid bacterium Lactobacillus acidophilus NCFM. Proc. Natl. Acad. Sci. USA.

[21] Makarova K., Slesarev A., Wolf Y., Sorokin A., Mirkin B., Koonin E., Pavlov A., Pavlova N., Karamychev V., Polouchine N. (2006). Comparative genomics of the lactic acid bacteria. Proc. Natl. Acad. Sci. USA.

[22] Ojala T., Kuparinen V., Koskinen J.P., Alatalo E., Holm L., Auvinen P., Edelman S., Westerlund-Wikström B., Korhonen T.K., Paulin L. (2010). Genome sequence of Lactobacillus crispatus ST1. J. Bacteriol..

[23] van de Guchte M., Penaud S., Grimaldi C., Barbe V., Bryson K., Nicolas P., Robert C., Oztas S., Mangenot S., Couloux A. (2006). The complete genome sequence of Lactobacillus bulgaricus reveals extensive and ongoing reductive evolution. Proc. Natl. Acad. Sci. USA.

[24] Toh H., Oshima K., Nakano A., Takahata M., Murakami M., Takaki T., Nishiyama H., Igimi S., Hattori M., Morita H. (2013). Genomic adaptation of the Lactobacillus casei group. PLoS ONE.

[25] Siezen R.J., Francke C., Renckens B., Boekhorst J., Wels M., Kleerebezem M., van Hijum S.A.F.T. (2012). Complete resequencing and reannotation of the Lactobacillus plantarum WCFS1 genome. J. Bacteriol..

[26] Kankainen M., Paulin L., Tynkkynen S., von Ossowski I., Reunanen J., Partanen P., Satokari R., Vesterlund S., Hendrickx A.P.A., Lebeer S. (2009). Comparative genomic analysis of Lactobacillus rhamnosus GG reveals pili containing a human- mucus binding protein. Proc. Natl. Acad. Sci. USA.

[27] Forde B.M., Neville B.A., O’Donnell M.M., Riboulet-Bisson E., Claesson M.J., Coghlan A., Ross R.P., O’Toole P.W. (2011). Genome sequences and comparative genomics of two Lactobacillus ruminis strains from the bovine and human intestinal tracts. Microb. Cell Fact..

[28] Reddy V.S., Saier M.H. (2012). BioV Suite--a collection of programs for the study of transport protein evolution. FEBS J..

[29] Ikeda M., Arai M., Lao D.M., Shimizu T. (2002). Transmembrane topology prediction methods: A re-assessment and improvement by a consensus method using a dataset of experimentally-characterized transmembrane topologies. In Silico Biol..

[30] Zhai Y., Saier M.H. (2001). A web-based program (WHAT) for the simultaneous prediction of hydropathy, amphipathicity, secondary structure and transmembrane topology for a single protein sequence. J. Mol. Microbiol. Biotechnol..

[31] Ito M., Hicks D.B., Henkin T.M., Guffanti A.A., Powers B.D., Zvi L., Uematsu K., Krulwich T.A. (2004). MotPS is the stator-force generator for motility of alkaliphilic Bacillus, and its homologue is a second functional Mot in Bacillus subtilis. Mol. Microbiol..

[32] Neville B.A., Forde B.M., Claesson M.J., Darby T., Coghlan A., Nally K., Ross R.P., O’Toole P.W. (2012). Characterization of pro-inflammatory flagellin proteins produced by Lactobacillus ruminis and related motile Lactobacilli. PLoS ONE.

[33] Payandeh J., Pfoh R., Pai E.F. (2013). The structure and regulation of magnesium selective ion channels. Biochim. Biophys. Acta.

[34] Pohland A.-C., Schneider D. (2019). Mg2+ homeostasis and transport in cyanobacteria—At the crossroads of bacterial and chloroplast Mg2+ import. Biol. Chem..

[35] Stockbridge R.B., Robertson J.L., Kolmakova-Partensky L., Miller C. (2013). A family of fluoride-specific ion channels with dual-topology architecture. Elife.

[36] Baida G.E., Kuzmin N.P. (1995). Cloning and primary structure of a new hemolysin gene from Bacillus cereus. Biochim. Biophys. Acta.

[37] Baida G.E., Kuzmin N.P. (1996). Mechanism of action of hemolysin III from Bacillus cereus. Biochim. Biophys. Acta.

[38] Ríos Colombo N.S., Chalón M.C., Navarro S.A., Bellomio A. (2018). Pediocin-like bacteriocins: New perspectives on mechanism of action and immunity. Curr. Genet..

[39] Ahn S.-J., Burne R.A. (2006). The atlA operon of Streptococcus mutans: Role in autolysin maturation and cell surface biogenesis. J. Bacteriol..

[40] Oppegård C., Rogne P., Emanuelsen L., Kristiansen P.E., Fimland G., Nissen-Meyer J. (2007). The two-peptide class II bacteriocins: Structure, production, and mode of action. J. Mol. Microbiol. Biotechnol..

[41] Sharma A., Srivastava S. (2014). Anti-Candida activity of two-peptide bacteriocins, plantaricins (Pln E/F and J/K) and their mode of action. Fungal Biol..

[42] Hanny E.L.L., Mustopa A.Z., Budiarti S., Darusman H.S., Ningrum R.A. (2019). Fatimah Efficacy, toxicity study and antioxidant properties of plantaricin E and F recombinants against enteropathogenic Escherichia coli K1.1 (EPEC K1.1). Mol. Biol. Rep..

[43] Maqueda M., Sánchez-Hidalgo M., Fernández M., Montalbán-López M., Valdivia E., Martínez-Bueno M. (2008). Genetic features of circular bacteriocins produced by Gram-positive bacteria. FEMS Microbiol. Rev..

[44] Saier M.H., Reddy B.L. (2015). Holins in bacteria, eukaryotes, and archaea: Multifunctional xenologues with potential biotechnological and biomedical applications. J. Bacteriol..

[45] Ahn S.-J., Qu M.-D., Roberts E., Burne R.A., Rice K.C. (2012). Identification of the Streptococcus mutans LytST two-component regulon reveals its contribution to oxidative stress tolerance. BMC Microbiol..

[46] Bayles K.W. (2003). Are the molecular strategies that control apoptosis conserved in bacteria?. Trends Microbiol..

[47] Brunskill E.W., Bayles K.W. (1996). Identification of LytSR-regulated genes from Staphylococcus aureus. J. Bacteriol..

[48] Tanouchi Y., Lee A.J., Meredith H., You L. (2013). Programmed cell death in bacteria and implications for antibiotic therapy. Trends Microbiol..

[49] Park J., Chun S., Bobik T.A., Houk K.N., Yeates T.O. (2017). Molecular Dynamics Simulations of Selective Metabolite Transport across the Propanediol Bacterial Microcompartment Shell. J. Phys. Chem. B.

[50] Crowley C.S., Sawaya M.R., Bobik T.A., Yeates T.O. (2008). Structure of the PduU shell protein from the Pdu microcompartment of Salmonella. Structure.

[51] Fàbrega A., Martin R.G., Rosner J.L., Tavio M.M., Vila J. (2010). Constitutive SoxS expression in a fluoroquinolone-resistant strain with a truncated SoxR protein and identification of a new member of the marA-soxS-rob regulon, mdtG. Antimicrob. Agents Chemother..

[52] Clancy J., Petitpas J., Dib-Hajj F., Yuan W., Cronan M., Kamath A.V., Bergeron J., Retsema J.A. (1996). Molecular cloning and functional analysis of a novel macrolide-resistance determinant, mefA, from Streptococcus pyogenes. Mol. Microbiol..

[53] Santagati M., Iannelli F., Cascone C., Campanile F., Oggioni M.R., Stefani S., Pozzi G. (2003). The novel conjugative transposon tn1207.3 carries the macrolide efflux gene mef(A) in Streptococcus pyogenes. Microb. Drug Resist..

[54] Schweikhard E.S., Ziegler C.M. (2012). Amino acid secondary transporters: Toward a common transport mechanism. Curr. Top. Membr..

[55] Burkovski A., Krämer R. (2002). Bacterial amino acid transport proteins: Occurrence, functions, and significance for biotechnological applications. Appl. Microbiol. Biotechnol..

[56] Buyuktimkin B., Zafar H., Saier M.H. (2019). Comparative genomics of the transportome of Ten Treponema species. Microb. Pathog..

[57] Teh A.H.T., Lee S.M., Dykes G.A. (2017). Identification of potential Campylobacter jejuni genes involved in biofilm formation by EZ-Tn5 Transposome mutagenesis. BMC Res. Notes.

[58] Kermani A.A., Macdonald C.B., Gundepudi R., Stockbridge R.B. (2018). Guanidinium export is the primal function of SMR family transporters. Proc. Natl. Acad. Sci. USA.

[59] Ioannou C.J., Hanlon G.W., Denyer S.P. (2007). Action of disinfectant quaternary ammonium compounds against Staphylococcus aureus. Antimicrob. Agents Chemother..

[60] Hvorup R.N., Winnen B., Chang A.B., Jiang Y., Zhou X.-F., Saier M.H. (2003). The multidrug/oligosaccharidyl-lipid/polysaccharide (MOP) exporter superfamily. Eur. J. Biochem..

[61] Tocci N., Iannelli F., Bidossi A., Ciusa M.L., Decorosi F., Viti C., Pozzi G., Ricci S., Oggioni M.R. (2013). Functional analysis of pneumococcal drug efflux pumps associates the MATE DinF transporter with quinolone susceptibility. Antimicrob. Agents Chemother..

[62] Kaatz G.W., DeMarco C.E., Seo S.M. (2006). MepR, a repressor of the Staphylococcus aureus MATE family multidrug efflux pump MepA, is a substrate-responsive regulatory protein. Antimicrob. Agents Chemother..

[63] Garvis S., Mei J.-M., Ruiz-Albert J., Holden D.W. (2002). Staphylococcus aureus svrA: A gene required for virulence and expression of the agr locus. Microbiology.

[64] Kaatz G.W., McAleese F., Seo S.M. (2005). Multidrug resistance in Staphylococcus aureus due to overexpression of a novel multidrug and toxin extrusion (MATE) transport protein. Antimicrob. Agents Chemother..

[65] Davidson A.L., Maloney P.C. (2007). ABC transporters: How small machines do a big job. Trends Microbiol..

[66] Paulsen I.T., Nguyen L., Sliwinski M.K., Rabus R., Saier M.H. (2000). Microbial genome analyses: Comparative transport capabilities in eighteen prokaryotes. J. Mol. Biol..

[67] Gibson G.R., Beatty E.R., Wang X., Cummings J.H. (1995). Selective stimulation of bifidobacteria in the human colon by oligofructose and inulin. Gastroenterology.

[68] Basavanna S., Khandavilli S., Yuste J., Cohen J.M., Hosie A.H.F., Webb A.J., Thomas G.H., Brown J.S. (2009). Screening of Streptococcus pneumoniae ABC transporter mutants demonstrates that LivJHMGF, a branched-chain amino acid ABC transporter, is necessary for disease pathogenesis. Infect. Immun..

[69] Wortham B.W., Patel C.N., Oliveira M.A. (2007). Polyamines in bacteria: Pleiotropic effects yet specific mechanisms. Adv. Exp. Med. Biol..

[70] Abrahams J.P., Leslie A.G., Lutter R., Walker J.E. (1994). Structure at 2.8 A resolution of F1-ATPase from bovine heart mitochondria. Nature.

[71] Balsera M., Buey R.M., Li X.-D. (2011). Quaternary structure of the oxaloacetate decarboxylase membrane complex and mechanistic relationships to pyruvate carboxylases. J. Biol. Chem..

[72] Kleerebezem M., Hols P., Bernard E., Rolain T., Zhou M., Siezen R.J., Bron P.A. (2010). The extracellular biology of the lactobacilli. FEMS Microbiol. Rev..

[73] Watanabe M., van der Veen S., Nakajima H., Abee T. (2012). Effect of respiration and manganese on oxidative stress resistance of Lactobacillus plantarum WCFS1. Microbiology.

[74] Morita H., Toh H., Fukuda S., Horikawa H., Oshima K., Suzuki T., Murakami M., Hisamatsu S., Kato Y., Takizawa T. (2008). Comparative genome analysis of Lactobacillus reuteri and Lactobacillus fermentum reveal a genomic island for reuterin and cobalamin production. DNA Res..

[75] Black P.N., DiRusso C.C. (2007). Vectorial acylation: Linking fatty acid transport and activation to metabolic trafficking. Novartis Found. Symp..

[76] ZJia Z., Pei Z., Maiguel D., Toomer C.J., Watkins P.A. (2007). The fatty acid transport protein (FATP) family: Very long chain acyl-CoA synthetases or solute carriers?. J. Mol. Neurosci..

[77] Davis J.K. (2012). Combining polysaccharide biosynthesis and transport in a single enzyme: Dual-function cell wall glycan synthases. Front. Plant Sci..

[78] Ernst C.M., Peschel A. (2011). Broad-spectrum antimicrobial peptide resistance by MprF-mediated aminoacylation and flipping of phospholipids. Mol. Microbiol..

[79] Brooijmans R., de Vos W.M., Hugenholtz J. (2009). Electron transport chains of lactic acid bacteria—Walking on crutches is part of their lifestyle. F1000 Biol. Rep..

[80] Akiyama Y. (2009). Quality control of cytoplasmic membrane proteins in Escherichia coli. J. Biochem..

[81] Coulter S.N., Schwan W.R., Ng E.Y., Langhorne M.H., Ritchie H.D., Westbrock-Wadman S., Hufnagle W.O., Folger K.R., Bayer A.S., Stover C.K. (1998). Staphylococcus aureus genetic loci impacting growth and survival in multiple infection environments. Mol. Microbiol..

[82] Steeb B., Claudi B., Burton N.A., Tienz P., Schmidt A., Farhan H., Mazé A., Bumann D. (2013). Parallel exploitation of diverse host nutrients enhances Salmonella virulence. PLoS Pathog..

[83] Costliow Z.A., Degnan P.H. (2017). Thiamine Acquisition Strategies Impact Metabolism and Competition in the Gut Microbe Bacteroides thetaiotaomicron. mSystems.

[84] Paulsen I.T., Sliwinski M.K., Saier M.H. (1998). Microbial genome analyses: Global comparisons of transport capabilities based on phylogenies, bioenergetics, and substrate specificities. J. Mol. Biol..

[85] Duar R.M., Lin X.B., Zheng J., Martino M.E., Grenier T., Perez-Munoz M.E., Leulier F., Ganzle M., Walter J. (2017). Lifestyles in transition: Evolution and natural history of the genus Lactobacillus. FEMS Microbiol. Rev..

[86] Castro-González J.M., Castro P., Sandoval H., Castro-Sandoval D. (2019). Probiotic Lactobacilli Precautions. Front. Microbiol..

[87] de Jong A., van Hijum S.A.F.T., Bijlsma J.J.E., Kok J., Kuipers O.P. (2006). BAGEL: A web-based bacteriocin genome mining tool. Nucleic Acids Res..

[88] Leyva Salas M., Thierry A., Lemaître M., Garric G., Harel-Oger M., Chatel M., Lê S., Mounier J., Valence F., Coton E. (2018). Antifungal Activity of Lactic Acid Bacteria Combinations in Dairy Mimicking Models and Their Potential as Bioprotective Cultures in Pilot Scale Applications. Front. Microbiol..

[89] Karami S., Roayaei M., Zahedi E., Bahmani M., Mahmoodnia L., Hamzavi H., Rafieian-Kopaei M. (2017). Antifungal effects of Lactobacillus species isolated from local dairy products. Int. J. Pharm. Investig..

[90] Gerbaldo G.A., Barberis C., Pascual L., Dalcero A., Barberis L. (2012). Antifungal activity of two Lactobacillus strains with potential probiotic properties. FEMS Microbiol. Lett..

[91] Kim S.W., Ha Y.J., Bang K.H., Lee S., Yeo J.-H., Yang H.-S., Kim T.-W., Lee K.P., Bang W.Y. (2020). Potential of Bacteriocins from Lactobacillus taiwanensis for Producing Bacterial Ghosts as a Next Generation Vaccine. Toxins.

[92] Lorca G.L., Barabote R.D., Zlotopolski V., Tran C., Winnen B., Hvorup R.N., Stonestrom A.J., Nguyen E., Huang L.-W., Kim D.S. (2007). Transport capabilities of eleven gram-positive bacteria: Comparative genomic analyses. Biochim. Biophys. Acta.

[93] Moreno-Hagelsieb G., Vitug B., Medrano-Soto A., Saier M.H. (2017). The Membrane Attack Complex/Perforin Superfamily. J. Mol. Microbiol. Biotechnol..

